# Prescribing Practice of Citalopram and Escitalopram in Older Adults Following the 2014 MHRA Guidance of Associated QTc Prolongation: A Clinical Audit

**DOI:** 10.1192/bjo.2024.588

**Published:** 2024-08-01

**Authors:** Shreyan Kar, Aparna Prasanna

**Affiliations:** 1 The Royal Wolverhampton NHS Trust, Wolverhampton, United Kingdom; 2 Black Country Healthcare NHS Foundation Trust, Wolverhampton, United Kingdom

## Abstract

**Aims:**

Citalopram and escitalopram are commonly used serotonin-specific reuptake inhibitors (SSRIs) for the treatment of depression and anxiety. These medications are known to cause corrected QT interval (QTc) prolongation, with risks of further arrhythmias. In 2014, the Medicines Healthcare Regulatory Agency (MHRA) published guidance outlining this risk and advised decreased maximum daily doses of citalopram 20mg and escitalopram 10mg in the elderly population. The aim of this audit was to explore the prescribing patterns of citalopram and escitalopram in a community sample of older adults with psychiatric disorders, against MHRA guidance.

**Methods:**

Older adults (aged >65 years) in the community mental health services in Wolverhampton, who were prescribed citalopram or escitalopram, were identified through a search of clinic letters in June 2023. We checked the medications, doses, history of QTc prolongation, concurrent medications that may prolong QTc, electrocardiogram (ECG) reviews, and any discussion about the risk. The data was collected by accessing the electronic patient record and related health records. In total 17 patients were included, with no exclusions.

**Results:**

Most of the patients (94.1%, n = 16) were on citalopram and only one patient was on escitalopram. The most common dose of citalopram was 20 mg (62.5%, 10/16), with one patient having a higher than the recommended dose (30 mg). Escitalopram was within the recommended dose. There was no history of QTc prolongation in any patient. Concurrent medications that could prolong QTc were identified in 35.3% (n = 6) of the patient population; all of these were antipsychotics. A small proportion (11.8%, n = 2) of the patients had documentation stating about QTc prolongation and arrhythmia risks for citalopram or escitalopram. A review of ECG when initiating or adjusting treatment was noted in only one patient.

**Conclusion:**

Most of the older adults had citalopram and escitalopram within recommended limits. A considerable proportion of patients had concurrent medications with an additional risk of prolonging QTc and subsequent arrhythmia. It is essential to consider ECG in all elderly patients before starting medications with a risk of QTc prolongation. There is a need to discuss the cardiac risk associated with citalopram and escitalopram with the patients and improve documentation. It may be better to provide written information to the patients and caregivers regarding this.

